# The Transcription Factor HAND1 Is Involved in Cortical Bone Mass through the Regulation of Collagen Expression

**DOI:** 10.3390/ijms21228638

**Published:** 2020-11-16

**Authors:** Noriko Funato, Yuki Taga, Lindsay E. Laurie, Chisa Tometsuka, Masashi Kusubata, Kiyoko Ogawa-Goto

**Affiliations:** 1 Department of Signal Gene Regulation, Tokyo Medical and Dental University (TMDU), 1-5-45 Yushima, Bunkyo-ku, Tokyo 113-8510, Japan; lindsay_preston@hotmail.com; 2 Research Core, Tokyo Medical and Dental University (TMDU), 1-5-45 Yushima, Bunkyo-ku, Tokyo 113-8510, Japan; 3 Nippi Research Institute of Biomatrix, 520-11 Kuwabara, Toride, Ibaraki 302-0017, Japan; y-taga@nippi-inc.co.jp (Y.T.); c-toometsuka@nippi-inc.co.jp (C.T.); qusubata@nippi-inc.co.jp (M.K.); kgoto@nippi-inc.co.jp (K.O.-G.)

**Keywords:** transcription factors, collagen, microRNAs, cortical bone, periosteum, X-ray microtomography

## Abstract

Temporal and/or spatial alteration of collagen family gene expression results in bone defects. However, how collagen expression controls bone size remains largely unknown. The basic helix-loop-helix transcription factor HAND1 is expressed in developing long bones and is involved in their morphogenesis. To understand the functional role of HAND1 and collagen in the postnatal development of long bones, we overexpressed *Hand1* in the osteochondroprogenitors of model mice and found that the bone volumes of cortical bones decreased in *Hand1^Tg/+^;Twist2-Cre* mice. Continuous *Hand1* expression downregulated the gene expression of type I, V, and XI collagen in the diaphyses of long bones and was associated with decreased expression of *Runx2* and *Sp7/Osterix*, encoding transcription factors involved in the transactivation of fibril-forming collagen genes. Members of the microRNA-196 family, which target the 3′ untranslated regions of *COL1A1* and *COL1A2*, were significantly upregulated in *Hand1^Tg/+^;Twist2-Cre* mice. Mass spectrometry revealed that the expression ratios of alpha 1(XI), alpha 2(XI), and alpha 2(V) in the diaphysis increased during postnatal development in wild-type mice, which was delayed in *Hand1^Tg/+^;Twist2-Cre* mice. Our results demonstrate that HAND1 regulates bone size and morphology through osteochondroprogenitors, at least partially by suppressing postnatal expression of collagen fibrils in the cortical bones.

## 1. Introduction

Skeletal dysplasia (osteochondrodysplasia) occurs in approximately 1 in 5000 births and is a major cause of severe short stature [1]. The size of each bone collectively determines the height and figure of the skeleton. Bone is primarily composed of a fibril-forming collagen matrix, accounting for approximately 80% of the bone extracellular matrix (ECM) [2]. Mutations in the collagen gene cause various skeletal dysplasias characterized by long bone deformities and midface hypoplasia, highlighting the importance of collagens in bone development [3]. Collagen is composed of three alpha chains that form a triple-helical structure which is essential for fibril strength and, bone collagens contribute to both the quantity and quality of the bone tissue [4]. Heterozygous mutations in the human *COL1A1* and *COL1A2* genes have been identified in patients with Ehlers–Danlos syndrome arthrochalasia type I (Online Mendelian Inheritance in Man [OMIM] #130060) and II (OMIM #617821), osteogenesis imperfecta (OMIM #166200, #166210, #259420, #166220), and Caffey disease (OMIM #114000). Heterozygous mutations in *COL5A1* and *COL5A2* have been identified in patients with Ehlers–Danlos syndrome classic type I (OMIM #130000) and II (OMIM #130010). Mutations in *COL11A1* and *COL11A2* are linked to Stickler syndrome type II (OMIM #604841), fibrochondrogenesis 1 (OMIM #228520), fibrochondrogenesis 2 (OMIM #614524), and Marshall syndrome (OMIM #154780). Type I collagen is the most abundant component of the bone matrix, accounting for up to 95% of bone collagens [2,5]. The cortical bone is composed of packed collagen type I fibrils and is highly mineralized, adding strength and rigidity to long bones [6]. The ratio of type V and XI collagens is approximately 3% of type I collagen in mature bones [5]. Type V and XI collagens assemble with type I collagen in the perichondrium of bones and modulate the size and shape of the fibrils [7,8,9], as well as interfering with the process of mineralization, which determines the stiffness of bones [6].

The long bone consists of the bony diaphysis, which connects the two ends, and the epiphyses. Cortical bone is the compact, dense outer layer that covers the bones, while trabecular bone lies in the interior of the epiphyses. Long bones develop through endochondral ossification in which the hyaline cartilage is replaced by mineralized tissue in the ossification center located in the middle of the diaphysis. Osteoblasts are differentiated from pluripotent perichondrial progenitor cells in response to osteogenic signaling inputs from differentiated chondrocytes [10,11,12]. Osteoblasts then invade the calcified cartilage with vascular tissues, secrete collagens, and create mineralized bone collar or cortical bone in the endochondral bones [13]. Collagen is involved in the formation of a tissue scaffold and structural stabilization, and also provides a substrate for cell anchorage, binds with non-collagenous proteins, and regulates the bioavailability of cytokines and growth factors [14]. The transcription factors RUNX2 (Runt-related transcription factor 2) and SP7/Osterix are essential for osteoblast specification and subsequent ossification of the bones [15,16,17]. *Runx2* is expressed in osteoblasts and perichondrial cells in the bone collar and in primary spongiosa in long bones, whereas *Sp7/Osterix* expression is restricted to osteoblasts [16,18]. *Runx2* and *Sp7/Osterix* encode transcription factors which regulate the expression of fibril-forming collagen genes [18,19,20,21]. *Runx2*- and *Sp7/Osterix*-deficient mice lack mineralization of endochondral and intramembranous bones due to the absence of functional osteoblasts [16,17]. In humans, *RUNX2* haploinsufficiency causes cleidocranial dysplasia (OMIM #119600), characterized by bone and teeth anomalies, including delayed fontanel closure, hypoplastic clavicles, short stature, and supernumerary teeth [22,23,24]. Homozygous mutations in *SP7/Osterix* have been linked to type XII osteogenesis imperfecta (OMIM #613849) characterized by skeletal anomalies including repeated bone fractures, generalized osteoporosis, and mild bone deformities.

Basic helix-loop-helix (bHLH) transcription factors play essential roles during embryonic development. *Hand1* and *Hand2*, which encode the bHLH transcription factor heart and neural crest derivatives expressed protein 1 (HAND1) and HAND2 respectively, are expressed in the developing limb primordium [25,26]. We previously reported that HAND1 and HAND2 act as negative regulators of intramembranous and endochondral ossification by inhibiting RUNX2 [25,27]. While conditional *Hand1* knockout mice with *Wnt1-Cre* or *Prrx1-Cre* exhibit no observable mandibular or limb abnormalities [26,28], mice that conditionally overexpress *Hand1* and *Hand2* with *Wnt1-Cre* or *Twist2-Cre* exhibit shortened and malformed mandible and limbs [25,27], indicating that the expression level of HAND transcription factors is critical for bone development. The overexpression of *HAND2* causes limb and heart defects in patients with 4q trisomy: dup (4)(q35.2-q31.22) [29,30]. However, the role of HAND transcription factors in bone size and quality control during postnatal growth remain largely unknown.

Here, we demonstrate that HAND1 plays a role in the regulation of the temporal expression of collagens involved in the postnatal development of long bone defects. In *Hand1*-overexpressing mice, the expression levels of fibril-forming collagens, as well as *Runx2* and *Sp7/Osterix*, the upstream genes of fibril-forming collagens, were significantly decreased. In addition, the expression levels of the members of the microRNA (miRNA)-196 family, which specifically target the 3′ untranslated regions (UTRs) of *COL1A1* and *COL1A2,* were significantly upregulated. Reduced expression of bone-related collagens in the cortical bones may result in long bone anomalies. These findings uncovered multifaceted roles of HAND1 in regulating bone size by negatively regulating the temporal expression of collagens in the cortical bone.

## 2. Results

### 2.1. Overexpression of Hand1 Induces Developmental Defects in the Skeletal Bones

**Figure 1:**
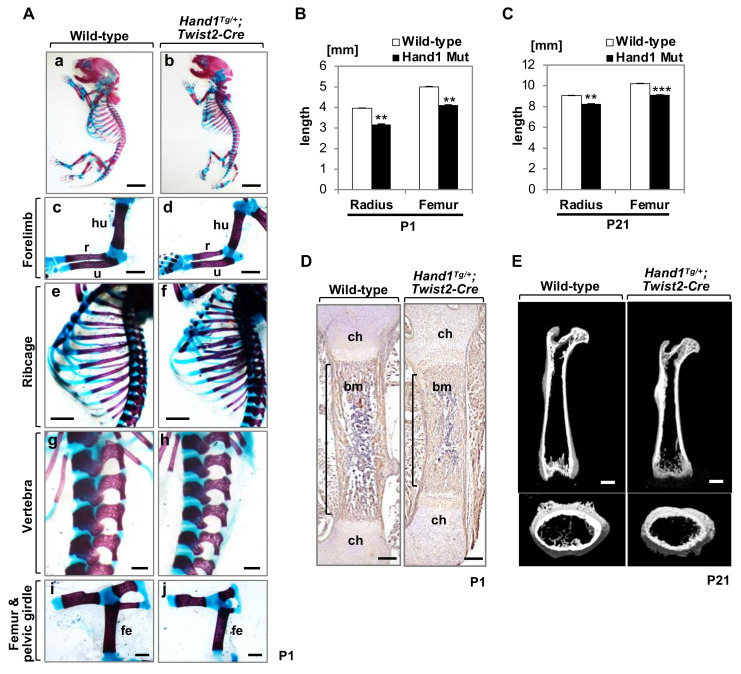
*Hand1* overexpression affects the morphology and size of long bones. (**A**) Staining of bones from wild-type and *Hand1*-overexpressing mice at postnatal day (P)1. hu, humerus; r, radius; u, ulna; fe, femur. Scale bars: 4 mm (**a**,**b**); 1 mm (**c**,**d**,**i**,**j**); 2 mm (**e**,**f**); 500 μm (**g**,**h**). (**B**,**C**) The lengths of the radius and femur bones of wild-type and *Hand1*-overexpressing mice at P1 (**B**) and P21 (**C**). The lengths of long bones from *Hand1*-overexpressing mice decreased compared to that of wild-type mice. *n* = 3, ** *p* < 0.01, *** *p* < 0.001. (**D**) Immunohistochemical staining for MMP13 in femurs from wild-type and *Hand1*-overexpressing mice at P1. Osteogenesis domain (shown in brackets) is hypoplastic in *Hand1*-overexpressing femurs. Scale bars: 200 μm. (**E**) Micro-computed tomography (micro-CT) analysis of femurs from wild-type and *Hand1*-overexpressing mice at postnatal day P21. Scale bars: 1 mm.

To investigate the role of HAND1 in the postnatal development of long bones, we examined mice that conditionally overexpressed *Hand1* (*Hand1^Tg/+^;Twist2-Cre*) driven by the *Twist2*-promoter [25,27]. During skeletal development, *Twist2* promoter-driven Cre expression is detected in the chondrocytes of the growth plate and in the osteoblasts in the periosteum, perichondrium, and endosteum without leakage in tendons or interlimb flanks [31,32,33]. All *Hand1*-overexpressing mice (*n* = 54) exhibited hypoplastic ossification of the skeletal bones, including long bones, ribcages, and vertebral bodies at P1 (Figure 1A), whereas *Hand1^Tg/+^* mice and *Twist2-Cre* mice were indistinguishable from their wild-type littermates. All (*n* = 54) *Hand1*-overexpressing mice also displayed preaxial polydactyly [25]. By P21, *Hand1*-overexpressing mice were severely dwarfed compared to littermate controls of the same sex [25]. The lengths of the femurs and radii of the *Hand1*-overexpressing mice decreased compared to that of the wild-type at P1 and P21 (Figure 1B,C). Matrix metallopeptidase 13 (MMP13), encoded by *MMP13*, is a downstream target of RUNX2 [34,35] and is exclusively expressed in primary ossification centers of the skeletal bones [36,37,38]. Immunostaining of MMP13 showed that the osteogenesis domain was hypoplastic in the *Hand1*-overexpressing femurs (Figure 1D). Micro-computed tomography (micro-CT) analysis demonstrated a reduction in the cortical bone volume (Figure 1E). These findings suggest that *Hand1* expression levels affect embryonic and postnatal development of skeletal bones and that the bone size alteration observed in *Hand1*-overexpressing mice continues during postnatal growth.

### 2.2. Micro-CT Analysis of Long Bones

**Figure 2:**
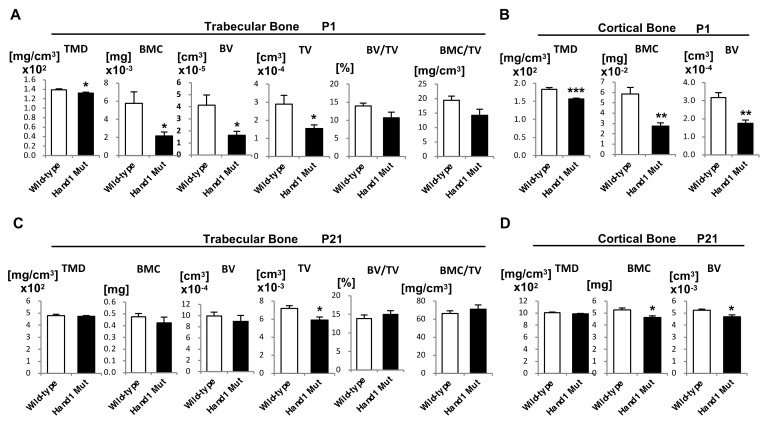
Micro-computed tomography (Micro-CT) analysis of femurs from *Hand1*-overexpressing mice. (**A**) Micro-CT analysis of trabecular bones from the femurs of wild-type and *Hand1*-overexpressing mice at postnatal day (P)1 (*n* = 3 per genotype). (**B**) Micro-CT analysis of the cortical bone of femurs from wild-type and *Hand1*-overexpressing mice at P1 (*n* = 3 per genotype). (**C**) Micro-CT analysis of trabecular bones of femurs from wild-type and *Hand1*-overexpressing mice at P21 (*n* = 3 per genotype). (**D**) Micro-CT analysis of cortical bones of femurs from wild-type and *Hand1*-overexpressing mice at P21 (*n* = 3 per genotype). Analysis of structural parameters of wild-type and *Hand1*-overexpressing mice indicated reduced cortical bone volume in the *Hand1*-overexpressing mice. * *p* < 0.05; ** *p* < 0.01; *** *p* < 0.001 (compared to the wild-type). TMD, bone tissue mineral density; BMC, bone mineral content; BV, bone volume; TV, total volume of interest; BV/TV, trabecular bone volume fraction; BMC/TV, volumetric bone mineral density.

*Hand1* overexpression affects the morphology and length of long bones (Figure 1). To assess the structure and mineral composition of long bones, we performed micro-CT scanning of the femurs from wild-type and *Hand1*-overexpressing mice at P1 and P21 and analyzed the trabecular and cortical bones. Micro-CT analysis demonstrated a significant reduction in bone mineral content (BMC), trabecular bone volume (BV), bone tissue mineral density (TMD; BMC/BV), the density measurement restricted to the calcified bone tissue only, and total volume of interest (TV) in the trabecular bones from P1 *Hand1*-overexpressing mice relative to their wild-type littermates (Figure 2A). In the cortical bones from P1 *Hand1*-overexpressing mice, TMD, BMC, and cortical BV were also significantly reduced (Figure 2B). In contrast, the skeletal anomalies displayed by P21 *Hand1*-overexpressing mice were not significantly different from wild-type littermates with respect to trabecular bone volume (BV; Figure 2C); however, a significant reduction was noted in total volume (TV; Figure 2C). In the cortical bones from P21 *Hand1*-overexpressing mice, BMC and cortical BV were significantly reduced (Figure 2D). These findings suggest that the coupling between bone formation and resorption is restored in the P21 *Hand1*-overexpressing mice and that *Hand1* has an inhibitory function in the postnatal development of cortical bone volume, but not in the maintenance of the trabecular bone volume.

### 2.3. Expression of Bone-Related Collagens in the Diaphyses

**Figure 3:**
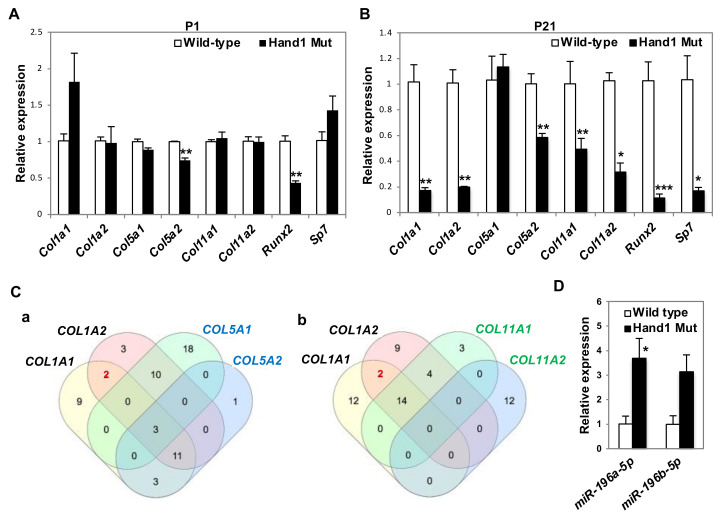
Expression of bone-related collagen genes are significantly decreased in the cortical bones of *Hand1*-overexpressing mice. (**A**,**B**) Real-time quantitative PCR analysis of bone-related collagen genes and their direct regulators in wild-type and *Hand1*-overexpressing mice (*n* = 3 per genotype) at postnatal (P) day 1 (**A**) and P21 (**B**). Expression of bone-related collagens, *Col1a1, Col1a2, Col5a2, Col11a1*, and *Col11a2,* and their regulators, *Sp7/Osterix* and *Runx2,* were significantly decreased in *Hand1*-overexpressing mice (Hand1 Mut) at P21. Data are represented as the mean ± standard error of mean (S.E.M). * *p* < 0.05; ** *p* < 0.01; *** *p* < 0.001 (compared to the wild-type). (**C**) (**a**) The number of putative miRNAs targeting collagen alpha chains (*COL1A1, COL1A2*, *COL5A1,* and *COL5A2*) are depicted in the Venn diagram using multiple list corporator (http://molbiotools.com). Two miRNAs (shown in red) commonly and specifically target *COL1A1* and *COL1A2.* (**b**) The number of putative miRNAs targeting collagen alpha chains (*COL1A1, COL1A2*, *COL11A1,* and *COL11A2*) are depicted in the Venn diagram. Two miRNAs (shown in red) commonly and specifically target *COL1A1* and *COL1A2.* Putative miRNAs that target collagen alpha chains are shown in Table S4. (**D**) Real-time quantitative PCR analysis of miR-196a and miR-196b in wild-type and *Hand1*-overexpressing mice (*n* = 3 per genotype) at P21. miR-196a was significantly upregulated in *Hand1*-overexpressing mice (Hand1 Mut). * *p* < 0.05 (compared to the wild-type).

Since *Hand1*-overexpressing mice display a failure in cortical bone formation, we focused our attention on cortical bone-related collagens. Collagen genes are involved in bone anomalies in various genetic diseases in humans (Tables S1 and S2). *Col1a1, Col1a2*, or *Col5a2* mutant mice exhibit a series of bone phenotypes, including decreased length of the long bones and thickness of the compact bones [39,40,41] (Table S3). When we investigated whether overexpression of *Hand1* affects the expression of collagen genes in the cortical bones of neonatal *Hand1*-overexpressing mice, we found that the expression of *Col5a2,* encoding the α2(V)-collagen chain, was significantly affected, whereas the expression of other collagen genes was unaffected (Figure 3A). In contrast, in P21 *Hand1*-overexpressing mice, the expression levels of *Col1a1, Col1a2, Col5a2, Col11a1*, and *Col11a2* were downregulated, whereas the expression of *Col5a1* barely changed (Figure 3B). *Runx2* and *Sp7/Osterix* encode transcription factors which regulate the expression of fibril-forming collagen genes [18,19,20,21]. Since the expression levels of collagen genes were downregulated in the *Hand1*-overexpressing mice (Figure 3A,B), we investigated whether overexpression of *Hand1* affects the expression of *Runx2* and *Sp7/Osterix* in the diaphysis. As expected, the expression of *Runx2* was significantly decreased in *Hand1*-overexpressing mice at P1 and P21 (Figure 3A,B). In addition, the expression of *Sp7/Osterix* was significantly decreased in *Hand1*-overexpressing mice at P21 (Figure 3B). These findings suggest that HAND1 regulates postnatal bone growth volume by regulating the temporal expression of cortical bone-related collagens.

### 2.4. miR-196 Are Upregulated in Hand1-Overexpressing Long Bones

Since *HAND1* significantly reduced *Col1a1 and Col1a2* expression in the diaphysis, we speculated that the expression of miRNAs specific to type I collagen genes may be deregulated in the long bones of *Hand1*-overexpressing mice. To identify the miRNAs specific to type I collagen genes, putative miRNAs that target collagen alpha chains (*Col1a1*, *Col1a2*, *Col5a1*, *Col5a2*, *Col11a1,* and *Col11a2*) were identified (Table S4) using the miRNA prediction software TargetScan 7.2. By depicting the number of putative microRNAs (miRNAs) targeting collagen alpha chains [(*COL1A1, COL1A2*, *COL5A1, COL5A2*) and (*COL1A1, COL1A2*, *COL11A1, COL11A2*)] in the Venn diagram (Figure 3C), we found two miRNAs, miR-196a and miR-196b, that commonly and specifically target *COL1A1* and *COL1A2* (Table S4). miR-196a is involved in the downregulation of type I collagen expression in scleroderma dermal fibroblasts [42]. The 3′ UTRs of both *Col1a1* and *Col1a2* were predicted targets of miR-196a-5p and miR-196b-5p, but not the 3′ UTR of other bone-related collagens (Figure 3C, Table S4). When we examined whether miR-196a-5p and miR-196b-5p were deregulated in *Hand1*-overexpressing mice, we found that the expression of these miRNAs was upregulated in *Hand1*-overexpressing mice compared to wild-type littermates (Figure 3D).

### 2.5. SDS-PAGE Analysis of Purified Collagen Samples

**Figure 4:**
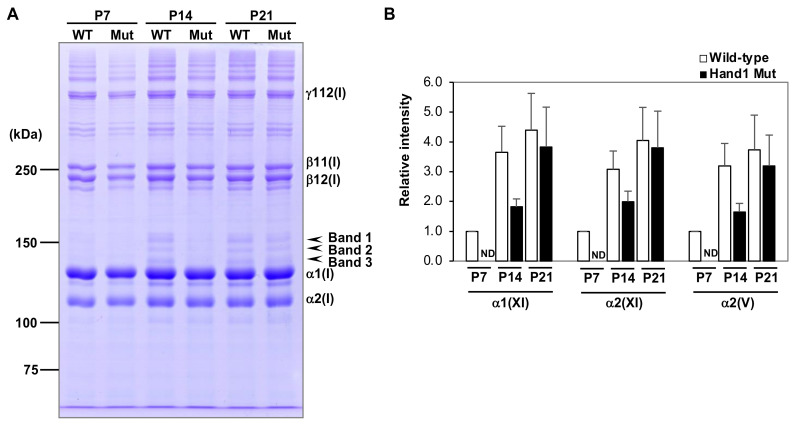
SDS-PAGE analysis of purified collagen samples. (**A**) The relative expression of collagen chains purified from the cortical bones from wild-type (WT) and *Hand1*-overexpressing (Mut) mice at postnatal day 7 (P7), P14, and P21 was evaluated by SDS-PAGE. The gel bands 1–3 (arrowheads) were identified as alpha 1(XI), alpha 2(XI), and alpha 2(V), respectively (Table S5). The expression of alpha 1(XI), alpha 2(XI), and alpha 2(V) (arrowheads) in the cortical bones of wild-type mice (WT) increased during postnatal development. This increase was delayed following continuous expression of *Hand1* in the osteochondral progenitors (Mut). Shown is a representative SDS-PAGE image from three independent experiments. (**B**) Changes in the levels of gel band intensity of alpha 1(XI), alpha 2(XI), and alpha 2(V) are represented as mean ± S.E.M. (*n* = 3). ND, not detected.

**Figure 5:**
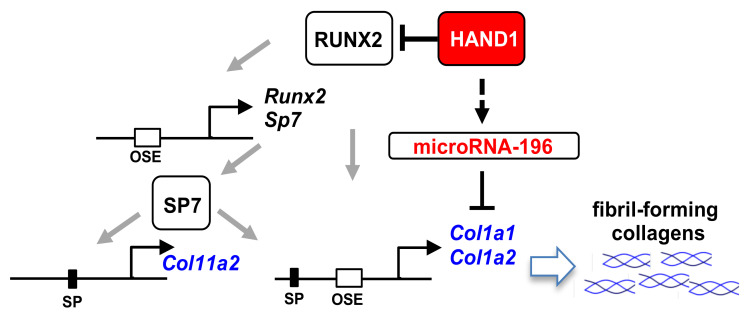
A predictive model for HAND1-mediated regulation of bone-related collagen expression. Expression of fibril-forming collagen genes (shown in blue) is decreased in the cortical bones of *Hand1*-overexpressing mice during postnatal development. HAND1 regulates osteoblast differentiation by inhibiting the transactivation of RUNX2 [25,27]. RUNX2 binds its own promoter and establishes a positive autoregulatory loop [44]. RUNX2 also acts upstream of *Col1a1, Col1a2*, and *Sp7/Osterix*. SP7/Osterix in turn directly upregulates *Col1a1, Col1a2,* and *Col11a2* by binding to Sp1 sites [18,19,20,21,45,46]. The expression levels of members of miR-196 family, which target the 3′ UTR of *Col1a1* and *Col1a2* [42], are upregulated in *Hand1*-overexpressing mice. OSE, RUNX2-binding site; SP, Sp1 binding site.

To further analyze whether HAND1 is functionally involved in the expression of collagens, thereby contributing to bone development, collagens were extracted and purified from the cortical bone from long bones at P7, P14, and P21. Equal amounts of collagen protein samples were loaded onto gels and analyzed by SDS-PAGE to evaluate the relative ratios of the collagen chains, not representing the absolute amount of each collagen type in bone tissue (Figure 4A). The bands of type I collagen were assigned based on their migration patterns (Figure 4A). Mass spectrometric analysis following in-gel trypsin digestion revealed that the trace bands above the alpha 1(I) chain band were type V and XI collagen alpha chains (Table S5; bands 1–3). Since type V and XI collagen chains were extracted first and more efficiently compared to type I collagen [43], the predominant extraction from bone by pepsin digestion resulted in detectable amounts of these minor collagens. The relative amounts of type V and XI collagens in the diaphyses increased with the maturation of long bones in the wild-type mice (Figure 4A,B). In contrast, the increase in type V and XI collagens was delayed by the continuous overexpression of *Hand1* in osteochondral progenitors (Figure 4A,B). We also investigated the possibility of an abnormal posttranslational modification of type I collagen, which may be associated with a significant reduction in the size of cortical bones in *Hand1*-overexpressing mice at P21, but the analysis did not indicate any significant change in the modifications of proline and lysine residues compared to the wild-type (data not shown). Collectively, our findings suggest that *Hand1* expression levels affect the postnatal bone volume of cortical bones through the temporal expression of bone-related collagens (Figure 5).

## 3. Discussion

In this study, by analyzing mouse models with *Hand1* overexpression in osteochondral progenitors, we demonstrated that HAND1 regulates postnatal bone growth volume by regulating the temporal expression of cortical bone-related collagens. The following findings support this conclusion: (1) overexpression of *Hand1* in osteochondral progenitors resulted in the reduction of cortical bone volume in long bones; (2) expression of bone-related collagen genes as well as *Runx2* and *Sp7/Osterix*, the upstream genes of fibril-forming collagens, decreased in *Hand1*-overexpressing mice; (3) expression of the members of miR-196 family, which target the 3′ UTR of *Col1a1* and *Col1a2*, was upregulated in *Hand1*-overexpressing mice; and (4) the increase in the postnatal expression of type V and XI collagens was delayed in the cortical bones of *Hand1*-overexpressing mice. The slower increase in type V and XI collagens during the maturation of long bones may be involved in the bone phenotypes observed in *Hand1*-overexpressing mice. While the bone phenotype was less apparent, the difference in the expression levels of fibril-forming collagen genes, *Runx2*, and *Sp7/Osterix* was significant between the *Hand1*-overexpressing mice and wild-type mice with increased postnatal days. The feedback from signaling pathways of endochondral bones [15,47], such as the parathyroid hormone-like hormone/parathyroid hormone-related protein, Indian hedgehog, Wnt/β-catenin, and fibroblast growth factor pathways as well as regulation by cilium assembly and transcription factors, may influence the bone phenotype of *Hand1*-overexpressing mice. We previously reported that Indian hedgehog (Ihh) expression was downregulated in the femur epiphyses of *Hand1*-overexpressing embryos, at least in part through the RUNX2-Ihh axis [25]. Aberrant expression of *Runx2* and *Ihh* may contribute to the abnormal development of cortical and trabecular bones. Since *Hand1*-overexpressing mice exhibit preaxial polydactyly [25] and a reduction in cortical bone volume, the *HAND1* region (5q33.2) and regulatory region of the *HAND1* gene might be the candidate regions involved in polydactyly and short stature in humans.

### 3.1. Type I Collagen Expression in Hand1-Overexpressing Mice

The ECM of bones is primarily composed of type I collagen [2]. Overexpression of *Hand1* in mice affects the expression of type I collagen genes, *Col1a1* and *Col1a2,* and results in a significant reduction in the cortical bone volume, suggesting that HAND1 acts as a regulator that determines the amount of type I collagen in long bones. Mice carrying *Col1a1* point mutations in the donor splice site of intron 36 or exon 9 exhibit decreased length of long bones and compact bone thickness [48]. Mice carrying *Col1a2* mutations in mouse models of human osteogenesis imperfecta also exhibit decreased bone volume, compact bone thickness, and abnormal compact bone morphology [41]. HAND1 regulates osteoblast differentiation by inhibiting transactivation of RUNX2 [25,27], which directly regulates its own expression in a positive autoregulatory loop [18]. RUNX2 is required for early stages of osteoblast differentiation and acts upstream of the fibril-forming collagens *Col1a1* and *Col1a2* [18,19]. Therefore, the expression of type I collagen genes may be regulated by HAND1 through the inhibition of RUNX2 transactivation. RUNX2 also acts upstream of SP7/Osterix [45], which in turn binds to the proximal promoters of *Col1a1* and *Col1a2* genes and upregulates these genes [20,21]. Since the 3′ UTRs of *Col1a1* and *Col1a2* have conserved target sites of miR-196a [42], and the expression levels of the members of miR-196 family were upregulated in *Hand1*-overexpressing mice, this family of miRNAs may play a role in determining the volume of cortical bones by regulating the expression of type I collagen genes. Interestingly, while members of the miR-196 family target only *Col1a1* and *Col1a2*, other bone-related collagen genes also have common miR-29 family-targeting sites in their 3′ UTRs. Furthermore, this property is conserved between mice and humans. Our findings suggest that in addition to HAND1-mediated downregulation of *Runx2* and *Sp7/Osterix* expression, HAND1-mediated upregulation of the members of miR-196 family may also contribute to the downregulation of type I collagen expression in the cortical bones.

### 3.2. Expression of Type V and XI Collagens in Hand1-Overexpressing Mice

*Hand1*-overexpressing mice exhibit decreased length of long bones with decreasing amounts of type V and XI collagen. Among the different members of fibrillar collagens, type V and XI collagens are minor but essential components of collagen fibrils by serving as templates for fibril polymerization of type I collagen [49]. Alpha 1(V), alpha 2(V), alpha 1(XI), and alpha 2(XI) chains accumulate in the collagen component of long bones at postnatal age [7]. Homozygous *Col5a2* knockout mice produced structurally abnormal type V and I collagen fibrils, and *Col5a2*-deficient femurs grow at a slower rate than control bones, resulting in decreased bone size [6]. Homozygous *Col11a1*-mutant mice also exhibit skeletal defects, including decreased length of long bones, abnormal hindlimb morphology, and micromelia [50]. An increased amount of type V collagen is observed in gracile bone dysplasia (OMIM #602361) [51]. These findings suggest that appropriate amounts of type V and XI collagens are critical in the development and morphogenesis of long bones downstream of HAND1. In *Hand1*-overexpressing mice, the expression of *Sp7/Osterix* was significantly decreased. SP7/Osterix upregulates *COL11A2* by binding to the GC-rich specific Sp1 binding site of the promoter [46]. Since the proximal promoters of *Col5a2* and *Col11a1* contain putative Sp1 binding sites [39,52], SP7/Osterix may also contribute in regulating the expression of type V and XI collagens in the cortical bones.

In summary, we found that the continuous expression of *Hand1* in osteochondral progenitors resulted in decreased expression of type I, V, and XI collagens in the diaphyses of long bones. In *Hand1*-overexpressing mice, the expression of *Runx2* and *Sp7/Osterix*, which encode transcription factors which regulate the expression of fibril-forming collagen genes, was significantly decreased. In addition, miR-196a-5p and miR-196b-5p, which target the 3′ UTRs of *COL1A1* and *COL1A2,* were upregulated in *Hand1*-overexpressing mice. Analysis of the fundamental relationship between the expression patterns of bone-related collagen genes responsible for bone structures will provide insights into how these collagen genes interact with factors involved in bone development. Investigation of the regulatory mechanisms of bone-related collagen gene expression by transcription factors and/or miRNAs may lead to new therapies for bone size and quality control.

## 4. Materials and Methods

### 4.1. Mice Conditionally Overexpressing Hand1

*Hand1*-overexpressing mice conditionally driven by *Twist2*-*Cre* (*Hand1^Tg/+^;Twist2-Cre*) have been described previously [25,53]. This study was carried out in strict accordance with the recommendations in the Guide for the Care and Use of Laboratory Animals of the National Institutes of Health. All animal experimental procedures were reviewed and approved by the Institutional Animal Care and Use Committee of the Tokyo Medical and Dental University (Permit Number: 0160215A, March 27, 2015).

### 4.2. Bone Staining, Histology, and Immunohistochemistry

Bone staining was performed using alizarin red and alcian blue as described previously [54]. Tissue samples for histology were fixed in 4% paraformaldehyde, decalcified, and embedded in paraffin, as described previously [25]. For MMP13 immunostaining, tissue sections were treated with 1mg/mL hyaluronidase (Sigma-Aldrich, St. Louis, MO, USA) at 37 °C for 45 min, and incubated with anti-MMP13 antibody (ab84594; Abcam, Cambridge, UK), followed by sequential treatment with components of the Vectastain Elite ABC kit (Vector Laboratories, Burlingame, CA, USA) and Immpact™ DAB peroxidase substrate (Vector Laboratories). The sections were counterstained with methyl green nuclear counterstain (Vector Laboratories).

### 4.3. Micro-Computed Tomography

Mineralized tissue formation was assessed by micro-computed tomography (micro-CT). Femurs were harvested at postnatal days 1 (P1) and P21 (*n* = 3 per group). Micro-CT images were scanned at a voltage of 75 kV and 140 μA in beam current, with filtration through a 0.1 mm brass plate, using an inspeXio SMX-100CT (Shimadzu, Kyoto, Japan). Scans were set at a pixel size of 512 × 512 and voxel size of 0.016 mm/voxel. The results were further analyzed using the TRI-3D-BON imaging system (Ratoc, Tokyo, Japan).

### 4.4. Real-Time Quantitative PCR

Bony diaphyses were harvested, frozen, wrapped in foil, and ground into a powder using a mortar and pestle containing liquid nitrogen. Total RNA containing the miRNA fraction was extracted using TRIzol (Thermo Fisher Scientific, Waltham, MA, USA). Real-time quantitative PCR (qRT-PCR) was performed as described previously [25] and the relative expression of the target genes was normalized to β-actin. For each sample, three replicates were run for each gene. Primer sequences used for qRT-PCR are listed in Table S6.

The miRNA fraction was extracted from total RNA using the miRNeasy Kit (Qiagen, Hilden, Germany) according to the manufacturer’s instructions. Putative miRNAs that target the collagen family (*Col1a1*, *Col1a2*, *Col5a1*, *Col5a2*, *Col11a1,* and *Col11a2*) were identified using the miRNA prediction software TargetScan 7.2 (http://www.targetscan.org) and are shown in Table S4. qRT-PCR of the miRNAs was performed using specifically designed stem-loop primers for mature miRNA analysis (GeneCopoeia, Rockville, MD, USA), following the manufacturer’s protocol. cDNA was synthesized from 2 μg of total RNA and specific qRT-PCR experiments for miRNAs were carried out using All-in-One miRNA qRT-PCR Detection Kit (GeneCopoeia). Amplification and detection were performed using the StepOne Plus System (Thermo Fisher Scientific). Each PCR reaction was run in triplicate. The endogenous control, U6 (GeneCopoeia) was used for normalization, and the relative expression of miRNAs was calculated using the 2^−ΔΔCt^ method.

### 4.5. Extraction and Purification of Cortical Bone Collagens

The diaphyses of the long bones were dissected from *Hand1*-overexpressing mice at P7, P14, and P21 and pooled prior to analysis. The bones were demineralized in 0.5 M EDTA (pH 7.8) for 3 d at 4 °C, and the soft tissue and bone marrow were removed from the diaphysis after cutting off the epiphyses. The demineralized bones were treated with 5 mg/mL pepsin (Sigma-Aldrich) in 0.5 M acetic acid for 3 d at 20 °C, and the extracted collagens were purified using salt precipitation (1 M NaCl) and isoelectric precipitation (pH 8.0).

### 4.6. Protein Identification Using in-Gel Digestion Followed by Mass Spectrometry

Protein identification was performed using in-gel digestion as described previously [55]. The relative expression of collagen chains purified from the cortical bones from wild-type and *Hand1*-overexpressing mice was evaluated by sodium dodecyl sulfate-polyacrylamide gel electrophoresis (SDS-PAGE) using a 5% gel under non-reducing conditions. After staining with Coomassie Brilliant Blue R-250, the SDS-PAGE gel was scanned and the band intensity was measured by densitometric analysis using Multi Gauge version 3.0 (Fujifim, Tokyo, Japan). Protein bands of purified collagen samples were excised and digested in-gel with trypsin (Promega, Madison, WI, USA) at 37 °C for 16 h. The tryptic digests were analyzed by liquid chromatography–mass spectrometry on a maXis II quadrupole time-of-flight mass spectrometer (Bruker Daltonics, Bremen, Germany) coupled to a Shimadzu Prominence UFLC-XR system (Shimadzu, Kyoto, Japan) with chromatographic separation using an Ascentis Express C18 HPLC column (2.7 μm particle size, L × I.D. 150 mm × 2.1 mm; Supelco, Bellefonte, PA, USA) as described previously [56]. A database search was performed against the UniProtKB/Swiss-Prot database (release 2018_05) for *Mus musculus* species (16970 protein entries) using ProteinPilot software 4.0 (AB Sciex, Foster City, CA, USA).

### 4.7. Statistical Analysis

Calculations and statistical analyses were performed using Microsoft Office Excel 2004 (Microsoft Corporation, Redmond, WA, USA). All data were expressed as mean ± standard error of mean (S.E.M). A two-tailed Student’s *t*-test was performed to compare two groups of independent samples and a normal distribution was assumed. Results with *p* < 0.05 were considered as statistically significant.

## Supplementary Materials

Supplementary materials can be found at https://www.mdpi.com/1422-0067/21/22/8638/s1. Table S1. Skeletal disorders induced by mutations in type I collagen genes in humans. Table S2. Skeletal disorders induced by mutations in types V and XI collagen genes in humans. Table S3. Skeletal phenotypes induced by mutations in cortical bone-related collagen genes in mice. Table S4. MicroRNAs that are predicted to target cortical bone-related collagen genes. Table S5. Summary of the proteins identified in bands that decreased in *Hand1*-overexpressing mice. Table S6. Primer sequences for real-time quantitative PCR.

## Abbreviations

BMC	Bone mineral content
BMC/TV	Volumetric bone mineral density
	
BV	Bone volume
ECM	Extracellular matrix
HAND1	Heart and neural crest derivatives expressed protein 1
MMP13	Matrix metallopeptidase 13
Micro-CT	Micro-computed tomography
OMIM	Online Mendelian Inheritance in Man
P	Postnatal day
S.E.M	Standard error of mean
SDS-PAGE	Sodium dodecyl sulfate-polyacrylamide gel electrophoresis
TMD	Bone tissue mineral density
TV	Total volume of interest
UTR	Untranslated regions
bHLH	Basic helix-loop-helix
miRNA	MicroRNA
qRT-PCR	Real-time quantitative PCR
