# Supplementary material for: The Transcription Factor HAND1 Is Involved in Cortical Bone Mass through the Regulation of Collagen Expression
Source: Int J Mol Sci. 2020 Nov 16;21(22):8638. doi: 10.3390/ijms21228638 (PMC7697595; doi:10.3390/ijms21228638)
Supplement: Supplementary file 1 [file ijms-21-08638-s001.pdf]

Supplemental Material

The transcription factor HAND1 is involved in cortical bone mass through the regulation of collagen expression

Noriko Funato *, Yuki Taga, Lindsay E. Laurie, Chisa Tometsuka,Masashi Kusubata, Kiyoko Ogawa-Goto

Table S1. Skeletal disorders induced by mutations in type I collagen genes in humans

Human					
**Gene Symbol**	**Protein**	**Disorder**	**Bone phenotypes in limbs**	**OMIM #**	**Inheritance**
COL1A1	COLLAGEN, TYPE I, ALPHA-1	Caffey disease<br>(R836C mutation)	- Curved tibia<br>- Cortical hyperostosis<br>- Irregularity of bone cortex	114000	AD
		Ehlers-Danlos syndrome, arthrochalasia type, 1	- Short stature<br>- Premature osteoarthritis<br>- Osteopenia<br>- Fractures	130060	AD
		Osteogenesis imperfecta, type I	- Mild osteopenia<br>- Varying degree of multiple fractures<br>- Wormian bones	166200	AD
		Osteogenesis imperfecta, type II	- Short limb dwarfism<br>- Numerous multiple fractures<br>- Wormian bones<br>- Soft calvaria<br>- Absent calvarial mineralization<br>- Large fontanelles<br>- Platyspondyly<br>- Tibial bowing<br>- Broad crumpled long bones<br>- Telescoped femur	166210	AD
		Osteogenesis imperfecta, type III	- Short limb dwarfism<br>- Short stature<br>- Wormian bones<br>- Large anterior fontanelle<br>- Undermineralized calvarium<br>- Severe, generalized Osteoporosis<br>- Multiple fractures present at birth<br>- Long bone deformity<br>- Bowing of limbs due to multiple fractures<br>- Thin gracile long bones<br>- Short deformed femurs<br>- Tibial bowing	259420	AD
		Osteogenesis imperfecta, type IV	- Short stature<br>- Mild-moderate skeletal deformity<br>- Varying degree of multiple fractures<br>- Wormian bones<br>- Scoliosis<br>- Kyphosis<br>- Biconcave flattened vertebrae<br>- Femoral bowing<br>- Bowed limbs due to multiple fractures	166220	AD
COL1A2	COLLAGEN, TYPE I, ALPHA-2	Ehlers-Danlos syndrome, arthrochalasia type, 2	- Fractures	617821	AD
		Ehlers-Danlos syndrome, cardiac valvular type	n.r.	225320	AR
		Osteogenesis imperfecta, type II	- Short limb dwarfism<br>- Numerous multiple fractures<br>- Wormian bones<br>- Soft calvaria<br>- Absent calvarial mineralization<br>- Large fontanelles<br>- Platyspondyly<br>- Tibial bowing<br>- Broad crumpled long bones<br>- Telescoped femur	166210	AD
		Osteogenesis imperfecta, type III	- Short limb dwarfism<br>- Short stature<br>- Wormian bones<br>- Large anterior fontanelle<br>- Undermineralized calvarium<br>- Severe, generalized Osteoporosis<br>- Multiple fractures present at birth<br>- Long bone deformity<br>- Bowing of limbs due to multiple fractures<br>- Thin gracile long bones<br>- Short deformed femurs<br>- Tibial bowing	259420	AD
		Osteogenesis imperfecta, type IV	- Short stature<br>- Mild-moderate skeletal deformity<br>- Varying degree of multiple fractures<br>- Wormian bones<br>- Scoliosis<br>- Kyphosis<br>- Biconcave flattened vertebrae<br>- Femoral bowing<br>- Bowed limbs due to multiple fractures	166220	AD

n.r., not reported; AD, autosomal dominant; AR, autosomal recessive.

Table S2. Skeletal disorders induced by mutations in types V and XI collagen genes in humans

Human					
**Gene Symbol**	**Protein**	**Disorder**	**Bone phenotypes in limbs**	**OMIM #**	**Inheritance**
COL5A1	COLLAGEN, TYPE V, ALPHA-1	Ehlers-Danlos syndrome, classic type, 1	- Short stature<br>- Osteoarthritis	130000	AD
COL5A2	COLLAGEN, TYPE V, ALPHA-2	Ehlers-Danlos syndrome, classic type, 2	n.r.	130010	AD
COL5A3	COLLAGEN, TYPE V, ALPHA-3	n.r.	n.r.	n.r.	n.r.
COL11A1	COLLAGEN, TYPE XI, ALPHA-1	Fibrochondrogenesis 1	- Short stature<br>- Widely patent coronal suture<br>- Widely patent sagittal suture<br>- Rhizomelic limb shortening<br>- Short, broad tubular bones<br>- Short fibulae	228520	AR
		Marshall syndrome	- Short stature<br>- Calvarial thickening<br>- Absent frontal sinuses<br>- Small, irregular distal femoral epiphyses<br>- Small, irregular proximal tibial epiphyses<br>- Outward radial bowing<br>- Outward ulnar bowing	154780	AD
		Stickler syndrome, type II	- Mild spondyloepiphyseal dysplasia<br>- Slender extremities	604841	AD
COL11A2	COLLAGEN, TYPE XI, ALPHA-2	Deafness, autosomal dominant 13	n.r.	601868	AD
		Deafness, autosomal recessive 53	n.r.	609706	AR
		Fibrochondrogenesis 2	- Relatively large skull<br>- Shortening of the long bones<br>- Widened metaphyses	614524	AD, AR
		Otospondylomegaepiphyseal dysplasia, autosomal dominant	- Epiphyseal dysplasia<br>- Premature osteoarthritis<br>- Large epiphyses	184840	AD
		Otospondylomegaepiphyseal dysplasia, autosomal recessive	- Short stature<br>- Epiphyseal dysplasia<br>- Premature osteoarthritis<br>- Wide flat epiphyses<br>- Short long bones	215150	AR

n.r., not reported; AD, autosomal dominant; AR, autosomal recessive.

Table S3. Skeletal phenotypes induced by mutations in cortical bone-related collagen genes in mice

Mouse				
**Gene**	**Protein**	**Mutation type**	**Bone phenotypes**	**References**
Col1a1	COLLAGEN, TYPE I, ALPHA-1	G349C substitution<br>(A mouse model of human disease, osteogenesis imperfecta type 4)	- Decreased body size<br>- Abnormal cranium morphology<br>- Decreased bone mineralization<br>- Long bone fractures<br>- Rib fractures	***J Biol Chem*** 1999, 274: 37923-37931
		Four point mutations<br>(A mouse model of human disease, osteogenesis imperfecta type 2)	- Abnormal neurocranium morphology<br>- Decrease in calvarial mineralization<br>- Rib fractures<br>- Short vertebral body	***J Biol Chem*** 1999, 274: 37923-37931
		Single point mutation: T to C point mutation in the exon 9 splice donor site that leads to skipping of exon 9 and the 18 amino acids coded within.<br>(A mouse model of human disease, osteogenesis imperfecta type 4 and Ehlers-Danlos syndrome)	- Abnormal bone healing<br>- Abnormal olecranon morphology<br>- Decreased length of long bones<br>- Short femur<br>- Abnormal pelvic girdle bone morphology<br>- Abnormal vertebral column morphology<br>- Decrease in bone volume/tissue volume<br>- Decrease in cortical thickness<br>- Decreased bone trabecula number	***Bone*** 2015, 81: 400-406;<br>***J Bone Miner Res*** 2014, 29 (6) : 1412-1423
		Single point mutation: T to A transversion in the donor splice site of intron 36 (GT->GA)<br>(A mouse model of human disease, osteogenesis imperfecta)	- Abnormal femur morphology<br>- Abnormal tibia morphology<br>- Decreased length of long bones<br>- Abnormal bone collagen fibril morphology<br>- Decreased compact bone thickness<br>- Abnormal trabecular bone morphology<br>- Decreased bone mass<br>- Fragile skeleton	***Sci Rep.*** 2017,15;7(1):11717;<br>***MGI Direct Data Submission***, 2008, J:132554
Col1a2	COLLAGEN, TYPE I, ALPHA-2	G610C substitution<br>(A mouse model of human disease, osteogenesis imperfecta)	- Increased bone mineral density<br>- Decreased bone mineral content<br>- Decreased bone volume<br>- Decreased compact bone area<br>- Decreased compact bone thickness	***J Bone Miner Res*** 2010, 25: 247-261
		A deletion of a single G residue at position 3978 (Col1a2:NM_007743.3:c.3978del)<br>(A mouse model of human disease, osteogenesis imperfecta type 3)	- Abnormal compact bone morphology<br>- Decreased bone strength	***Bone*** 1996,19 (6): 575-579
Col5a1	COLLAGEN, TYPE V, ALPHA-1	Knockout<br>(A mouse model of human disease, type I Ehlers-Danlos syndrome)	- Embryonic lethal	***J Biol Chem*** 2004, 279: 53331-53337
Col5a2	COLLAGEN, TYPE V, ALPHA-2	Intragenic deletion<br>(A mouse model of human disease, type I Ehlers-Danlos syndrome)	n.r.	***Am J Pathol*** 2015, 185: 2000-2011
		Intragenic deletion	- Decreased body size<br>- Mutant bone (femur) grows at a slower rate than wild-type bone	***Nat Genet*** 1995, 9: 31-36
Col5a3	COLLAGEN, TYPE V, ALPHA-3	Intragenic deletion	n.r.	***J Clin Invest*** 2011, 121: 769-783
Col11a1	COLLAGEN, TYPE XI, ALPHA-1	Intragenic deletion<br>(A mouse model of human disease, Stickler syndrome)	- Micromelia<br>- Abnormal hindlimb morphology<br>- Short mandible<br>- Abnormal long bone diaphysis morphology<br>- Abnormal long bone metaphysis morphology<br>- Decreased length of long bones<br>- Increased diameter of long bones<br>- Abnormal trabecular bone morphology<br>- Abnormal epiphyseal plate morphology<br>- Decreased bone mineralization	***J Cell Biol*** 1971, 48: 580-593
Col11a2	COLLAGEN, TYPE XI, ALPHA-2	Intragenic deletion<br>(A mouse model of human disease, Stickler syndrome)	- Abnormal cranium morphology<br>- Disorganized long bone epiphyseal plate<br>- Abnormal articular cartilage morphology	***Dev Dyn*** 2001, 222: 141-152

n.r., not reported.

Table S4. MicroRNAs that are predicted to target cortical bone-related collagen genes

COL1A1	COL1A2	COL5A1	COL5A2	COL11A1	COL11A2
**hsa-miR-196a-5p**	**hsa-miR-196a-5p**				
**hsa-miR-196b-5p**	**hsa-miR-196b-5p**				
hsa-let-7a-5p	hsa-let-7a-5p	hsa-miR-181a-5p	hsa-let-7a-5p	hsa-let-7a-5p	hsa-miR-23a-3p
hsa-let-7b-5p	hsa-let-7b-5p	hsa-miR-181b-5p	hsa-let-7b-5p	hsa-let-7b-5p	hsa-miR-23b-3p
hsa-let-7c-5p	hsa-let-7c-5p	hsa-miR-181c-5p	hsa-let-7c-5p	hsa-let-7c-5p	hsa-miR-23c
hsa-let-7d-5p	hsa-let-7d-5p	hsa-miR-181d-5p	hsa-let-7d-5p	hsa-let-7d-5p	hsa-miR-125a-5p
hsa-let-7e-5p	hsa-let-7e-5p		hsa-let-7e-5p	hsa-let-7e-5p	hsa-miR-125b-5p
hsa-let-7f-5p	hsa-let-7f-5p		hsa-let-7f-5p	hsa-let-7f-5p	hsa-miR-128-3p
hsa-let-7g-5p	hsa-let-7g-5p		hsa-let-7g-5p	hsa-let-7g-5p	hsa-miR-130a-5p
hsa-let-7i-5p	hsa-let-7i-5p		hsa-let-7i-5p	hsa-let-7i-5p	hsa-miR-216a-3p
hsa-miR-29a-3p	hsa-miR-29a-3p	hsa-miR-29a-3p	hsa-miR-29a-3p	hsa-miR-29a-3p	hsa-miR-3681-3p
hsa-miR-29b-3p	hsa-miR-29b-3p	hsa-miR-29b-3p	hsa-miR-29b-3p	hsa-miR-29b-3p	hsa-miR-4319
hsa-miR-29c-3p	hsa-miR-29c-3p	hsa-miR-29c-3p	hsa-miR-29c-3p	hsa-miR-29c-3p	
hsa-miR-98-5p	hsa-miR-98-5p		hsa-miR-98-5p	hsa-miR-98-5p	
hsa-miR-129-5p					
hsa-miR-133a-3p.2					
hsa-miR-133b					
hsa-miR-143-3p			hsa-miR-143-3p		
hsa-miR-193a-5p					
hsa-miR-218-5p					
hsa-miR-338-3p					
hsa-miR-371a-5p					
hsa-miR-382-5p					
hsa-miR-532-3p					
hsa-miR-4500	hsa-miR-4500		hsa-miR-4500	hsa-miR-4500	
hsa-miR-4458	hsa-miR-4458		hsa-miR-4458	hsa-miR-4458	
hsa-miR-4770			hsa-miR-4770		
hsa-miR-6088			hsa-miR-6088		
	hsa-miR-7-5p				
	hsa-miR-19a-3p				
	hsa-miR-19b-3p				
	hsa-miR-25-3p	hsa-miR-25-3p			
	hsa-miR-26a-5p	hsa-miR-26a-5p		hsa-miR-26a-5p	
	hsa-miR-26b-5p	hsa-miR-26b-5p		hsa-miR-26b-5p	
	hsa-miR-32-5p	hsa-miR-32-5p			
	hsa-miR-92a-3p	hsa-miR-92a-3p			
	hsa-miR-92b-3p	hsa-miR-92b-3p			
	hsa-miR-363-3p	hsa-miR-363-3p			
	hsa-miR-367-3p	hsa-miR-367-3p			
	hsa-miR-1297	hsa-miR-1297		hsa-miR-1297	
	hsa-miR-4465	hsa-miR-4465		hsa-miR-4465	
		hsa-miR-27a-3p			hsa-miR-27a-3p
		hsa-miR-27b-3p			hsa-miR-27b-3p
		hsa-miR-31-5p			
		hsa-miR-135a-5p			
		hsa-miR-135b-5p			
		hsa-miR-137			
		hsa-miR-182-5p			
		hsa-miR-192-5p			
		hsa-miR-582-5p			
		hsa-miR-215-5p			
		hsa-miR-370-5p			
		hsa-miR-493-3p			
		hsa-miR-1193			
		hsa-miR-4262			
			hsa-miR-144-3p	hsa-miR-144-3p	
				hsa-miR-300	
				hsa-miR-381-3p	

Table S5. Summary of the proteins identified in bands that were decreased in *Hand1*-overexpressing mice

Band	N	Score	Coverage (%)	Accession number	Protein
1	1	12.55	23.3	sp|Q61245|COBA1_MOUSE	Collagen alpha-1(XI) chain
	2	7.46	22.2	sp|P11087|CO1A1_MOUSE	Collagen alpha-1(I) chain
	3	2.61	17.8	sp|Q01149|CO1A2_MOUSE	Collagen alpha-2(I) chain
2	1	10.23	34.5	sp|Q64739|COBA2_MOUSE	Collagen alpha-2(XI) chain
	5	0.95	19.9	sp|P11087|CO1A1_MOUSE	Collagen alpha-1(I) chain
3	1	15.93	29.2	sp|P11087|CO1A1_MOUSE	Collagen alpha-1(I) chain
	2	6.82	25.7	sp|Q3U962|CO5A2_MOUSE	Collagen alpha-2(V) chain

Gel bands 1–3 from the long bone at P21 (Figure 4) were analyzed by liquid chromatography–mass spectrometry (LC–MS) following in-gel digestion. Keratin was excluded from the list. Type I collagens are considered to be contaminants from the main bands (approximately 100–150 kDa) and are indicated in gray.

Table S6. Primer sequences for real-time quantitative PCR

Gene	Primer Sequence (Right)	Primer Sequence (Left)
Col1a1	5’-ACATGTTCAGCTTTGTGGACC-3’	5’-TAGGCCATTGTGTATGCAGC-3’
Col1a2	5’-GACTGTAAGAAGCGAGTTACC-3’	5’-GCCTTCAAAGACTTCATCG-3’
Col5a1	5’-AAGCGTGGGAAACTGCTCTCCTAT-3’	5’-AGCAGTTGTAGGTGACGTTCTGGT-3’
Col5a2	5’-AAAGCCCAGGAACAAGAGAA-3’	5’- CATGGAGAAGGTTTCCAAATG-3’
Col11a1	5’-TGGAATCATGGTATTTGGAACA-3’	5’-ATATGCTGCCTTGGGGTCTC-3’
Col11a2	5’-AGTCCCTTGCCATTCCTTG-3’	5’-GGGGGTCCCTCTACAAACAT-3’
Runx2	5’-GCTCACGTCGCTCATCTTG-3’	5’-TATGGCGTCAAACAGCCTCT-3’
Sp7	5’-CTCTCCATCTGCCTGACTCC-3’	5’-GGACTGGAGCCATAGTGAGC-3’
Actb	5’-ATGGAGGGGAATACAGCCC-3’	5’-TTCTTTGCAGCTCCTTCGTT-3’
